# SDF1 in the dorsal corticospinal tract promotes CXCR4+ cell migration after spinal cord injury

**DOI:** 10.1186/1742-2094-8-16

**Published:** 2011-02-16

**Authors:** Vicki M Tysseling, Divakar Mithal, Vibhu Sahni, Derin Birch, Hosung Jung, Richard J Miller, John A Kessler

**Affiliations:** 1 Northwestern University's Feinberg School of Medicine, Department of Neurology, Chicago, IL 60611, USA; 2 Northwestern University's Feinberg School of Medicine, Department of Molecular Pharmacology and Biological Chemistry, Chicago, IL 60611, USA

## Abstract

**Background:**

Stromal cell-derived factor-1 (SDF1) and its major signaling receptor, CXCR4, were initially described in the immune system; however, they are also expressed in the nervous system, including the spinal cord. After spinal cord injury, the blood brain barrier is compromised, opening the way for chemokine signaling between these two systems. These experiments clarified prior contradictory findings on normal expression of SDF1 and CXCR4 as well as examined the resulting spinal cord responses resulting from this signaling.

**Methods:**

These experiments examined the expression and function of SDF1 and CXCR4 in the normal and injured adult mouse spinal cord primarily using CXCR4-EGFP and SDF1-EGFP transgenic reporter mice.

**Results:**

In the uninjured spinal cord, SDF1 was expressed in the dorsal corticospinal tract (dCST) as well as the meninges, whereas CXCR4 was found only in ependymal cells surrounding the central canal. After spinal cord injury (SCI), the pattern of SDF1 expression did not change rostral to the lesion but it disappeared from the degenerating dCST caudally. By contrast, CXCR4 expression changed dramatically after SCI. In addition to the CXCR4+ cells in the ependymal layer, numerous CXCR4+ cells appeared in the peripheral white matter and in the dorsal white matter localized between the dorsal corticospinal tract and the gray matter rostral to the lesion site. The non-ependymal CXCR4+ cells were found to be NG2+ and CD11b+ macrophages that presumably infiltrated through the broken blood-brain barrier. One population of macrophages appeared to be migrating towards the dCST that contains SDF1 rostral to the injury but not towards the caudal dCST in which SDF1 is no longer present. A second population of the CXCR4+ macrophages was present near the SDF1-expressing meningeal cells.

**Conclusions:**

These observations suggest that attraction of CXCR4+ macrophages is part of a programmed response to injury and that modulation of the SDF1 signaling system may be important for regulating the inflammatory response after SCI.

## Background

Chemokines, originally described for their role in promoting the migration of leukocytes during inflammatory responses, have many well-established pro-inflammatory and pro-migratory roles in the immune system. More recently they have been found to have numerous important functions in the nervous system as well [1-3]. In the developing brain, neurons and glia express various chemokine receptors and are therefore potential targets for chemokine signaling [2,3]. For example, neonatal oligodendrocyte maturation and myelination, axonal growth, neuronal proliferation and neuronal survival are just a few of the normal processes regulated by chemokines and particularly by one chemokine pair, stromal cell-derived factor-1 (SDF-1; also known as CXCL12) with its receptor CXC chemokine receptor 4 (CXCR4) [4-11].

After spinal cord injury (SCI) the blood-brain barrier is compromised leading to a large infiltration of macrophages. Beneficial as well as deleterious effects have been ascribed to immune cells that infiltrate the nervous system after neural injury, including SCI [12-18]. Since cells of both the nervous and immune systems express SDF1 and CXCR4, chemokine signaling between these cells could regulate spinal cord responses to injury. However, due to contradictory reports of SDF1/CXCR4 signaling in the spinal cord and following SCI, their role remains unclear [19-22]. In the adult spinal cord GABAergic neurons [20] and corticospinal motor neurons [22] reportedly express CXCR4. Further, levels of SDF1 mRNA increase 7 days after SCI suggesting a possible role in injury responses [21]. We therefore sought to define the expression and possible function of SDF1 and its receptor, CXCR4 in the normal spinal cord and after SCI. We observed that in the normal spinal cord, SDF1 is expressed by the dorsal corticospinal tract (dCST) and by the meninges whereas ependymal cells express CXCR4. After SCI, infiltrating macrophages and perhaps ependymal cells, both of which express CXCR4, appear to migrate towards both the dCST and meningeal sources of SDF1. Another population of CXCR4+ cells remains in the ependymal layer after SCI. These observations suggest that this signaling system may help to regulate inflammatory responses after SCI, particularly in the dorsal spinal cord.

## Methods

### Animals

The transgenic mice used in this study (all kindly provided by Richard Miller, Northwestern University) were CXCR4-EGFP (CXCR4BAC::EGFP) and SDF-1-EGFP (SDF-1BAC::EGFP) from The Rockefeller University, New York, NY and The Gene Expression Nervous System Atlas (GENSAT) project, National Institute of Neurological Disorders and Stroke contract N01Nso2331 to The Rockefeller University, New York, NY http://www.gensat.org. All animal-related procedures were approved by the Northwestern University animal care and use committee.

### Spinal cord injury

Adult mice are anesthetized using inhalation anesthetic (2.5% isofluorane in 100% oxygen which was administered using VetEquip Rodent anesthesia machine). After laminectomy at the T10 vertebral segment, the spinal cord was compressed dorsoventrally by the extradural application of a 24 g modified aneurysm clip for 1 min (FEJOTA mouse clip). After SCI, the skin was sutured using AUTOCLIP (9 mm; BD Biosciences, San Jose, CA). Bladders were manually emptied twice daily throughout the duration of the study. Post surgery and in the event of discomfort, Meloxicam (0.3 mg/kg, s.c., once daily) was administered. Gentamycin was administered once daily in the event of hematuria (20 mg/kg) subcutaneously for 5 d.

### Dextran dye injection

Adult mice were anesthetized with Avertin and were injected with mini-ruby-conjugated BDA (Invitrogen) using a 10 μl Hamilton microsyringe fitted with a pulled-glass micropipette. The corticospinal tract was labeled with three injections (0.5 μl each) made at 1.0 mm lateral to the midline at 0.5 mm anterior, 0.5 mm posterior, and 1.0 mm posterior to bregma, and at a depth of 0.5 mm from the cortical surface. Animals were sacrificed 14 d after BDA injection and perfused.

### Animal perfusions and tissue acquisition

Animals were sacrificed using an overdose of carbon dioxide and transcardially perfused with PBS followed by 4% paraformaldehyde in PBS. The spinal cords were dissected and either fixed overnight in 30% sucrose in 4% PFA or fixed for 2 hours in 4% PFA and dehydrated overnight in 30% sucrose. The spinal cords were then frozen in Tissue-Tek embedding compound and sectioned on a Leica (Deerfield, IL) CM3050 S cryostat at 20 μm.

### Immunohistochemistry

Sections were rinsed with PBS thrice and then incubated with blocking solution (10% normal goat serum, 0.25% TritonX-100, 1% BSA in PBS) at room temperature for 1 hour. The sections were again rinsed thrice with PBS and incubated with primary antibody [(GFAP; 1:250; mouse monoclonal IgG1; Sigma), (CD11b; 1:200; rat monoclonal IgG2b; Serotec), (Nestin; 1:200; mouse monoclonal IgG1; Abcam), (NG2; 1:200; rabbit; Chemicon), (RC2; 1:8; mouse monoclonal IgM, DSHB) in blocking solution without NGS at 4 deg C overnight. The SDF1-EGFP and CXCR4-EGFP expression in these reporter mice was very strong and required no antibody amplification. After this, sections were rinsed thrice with PBS and incubated with Alexa Fluorconjugated secondary antibodies (1:500; Invitrogen, Carlsbad, CA) for 1 h at room temperature. Sections were finally rinsed thrice with PBS and then incubated with Hoechst nuclear stain in PBS for 10 minutes at room temperature. After a final rinse with PBS, they were mounted using Prolong Gold anti-fade reagent (Invitrogen) and imaged using a Zeiss (Thornwood, NY) UVLSM-Meta confocal microscope or Axiovert fluorescent microscope with the AxiocamHR camera.

## Results

### SDF1 and CXCR4 expression in the normal, uninjured spinal cord

**Figure 1:**
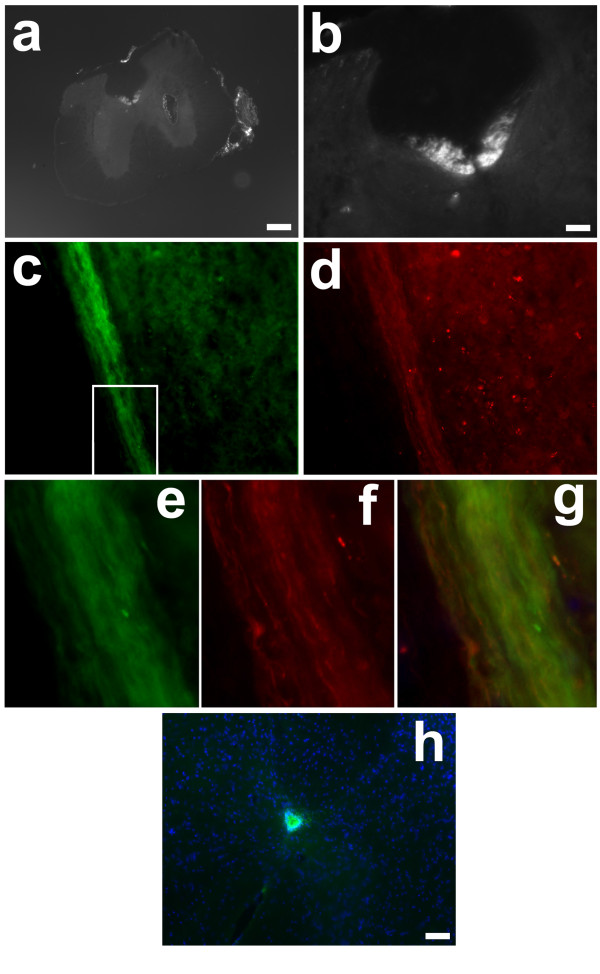
**SDF1 and CXCR4 expression in the normal spinal cord**. SDF1 is expressed throughout the dCST (a-g) a-b, Cross sections of SDF1-GFP mouse spinal cords show SDF1 in the meninges as well as in the dCST. Rhodamine labeled dextran injection into the sensorimotor cortex shows co-label of SDF1 and dextran dye confirming presence of SDF1-GFP in the dCST (c-g)(red:biotin, green:SDF1-EGFP, blue:Hoechst). e-g, Magnified images of the boxed area of c and d further demonstrating co-label of SDF1 and dCST label. h. The cognate receptor of SDF1, CXCR4, is found only in the ependymal layer (green:CXCR4-EGFP, blue:Hoechst). Scale bars a: 200 μm, b-d: 50 μm, h:20 μm

We first examined expression patterns of SDF1 and CXCR4 in the intact adult spinal cord using well described CXCR4-EGFP and SDF1-EGFP transgenic reporter mice [23,24]. In this as in the following experiments, the EGFP expression in these reporter mice was very strong and required no antibody amplification. SDF1 was strongly expressed by meningeal cells in the adult spinal cord (Figure 1a, 2a, c) analogous to findings in the brain [25]. Interestingly, however, SDF1 was also highly expressed in the dorsal funiculus in what appears to be the dorsal corticospinal tract (dCST) (Figure 1a-e). To confirm the localization in the dCST, BDA-labeled dye was injected into motor cortex to label the tract. SDF1-EGFP (Figure 1c) and BDA-labeled dye (Figure 1d) were colocalized to the same fibers (Figure 1e-g) confirming expression by dCST neurons. By contrast, CXCR4-EGFP+ cells were only found in the ependymal layer (Figure 1h). Therefore, in the adult uninjured spinal cord, SDF1 and CXCR4 are expressed in separate, nonadjacent regions.

**Figure 2:**
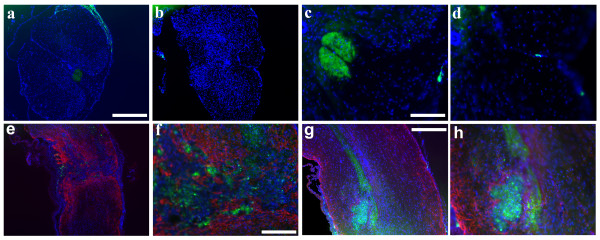
**5 weeks post SCI, SDF1 is found in the dCST rostral to the injury and inside the lesion epicenter**. Thoracic cross sections demonstrate SDF1-GFP in the dCST rostral (a, c), but not caudal (b, d) to the injury, however, the SDF1 is present in the meninges throughout (a-d)(green:SDF1-EGFP, blue:Hoechst). A longitudinal section that does not contain the dCST shows some migrating cells expressing SDF1, but most are trapped within the lesion epicenter by the GFAP+ scar (e, f). Longitudinal sections containing the dCST show the end of the tract at a cavitation noted by the high density of nuclei and the GFAP+ scar around its rim (g, h)(red:GFAP, green:SDF1-EGFP, blue:Hoechst) Scale bars: a-b, e: 200 μm, c-d, h: 50 μm, f: 25, g: 100 μm.

### SDF1 expression following SCI

To investigate SDF1expression following SCI, we used a severe clip compression model of SCI that produces a consistent injury in mice where an initial impact is followed by persistent compression analogous to most cases of human SCI [26,27]. At 5 weeks post SCI, SDF1 was still found in the meninges (Figure 2a) and in the dCST rostral to the lesion (Figure 2a, c). However SDF1 expression was no longer detectable in the area of the dCST caudal to the lesion site (Figure 2b, d). Longitudinal sections containing the dCST demonstrated the termination and dissipation of the tract at the lesion site (Figure 2gh). At the site of injury, SDF1 was also expressed by infiltrating cells that were largely restricted to the lesion epicenter by the glial scar (Figure 2ef).

### CXCR4 expression in the injured spinal cord

**Figure 3:**
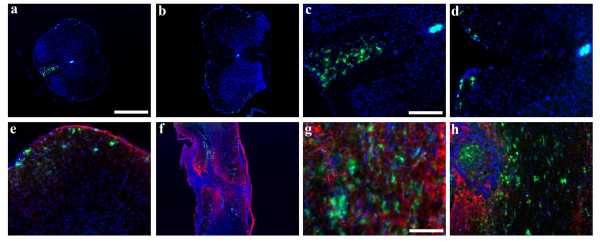
**5 weeks post SCI, CXCR4 is found in 3 different areas of the spinal cord**. Thoracic cross sections demonstrate CXCR4-GFP in the ependymal layer (a-d), peripherally toward the meninges (a-e) and in the dorsal funiculus rostral to the injury (a, c) but not caudal to it (b, d)(green:CXCR4-EGFP, blue:Hoechst). Longitudinal sections show CXCR4-GFP+ cells in the lesion epicenter as noted in f and in the cavitation in h, but also outside the lesion (f, h). Some CXCR4-GFP cells were found within the GFAP+ scar, but were distinct from the GFAP+ cells (g)(red:GFAP, green:CXCR4-EGFP, blue:Hoechst). Scale bars: a-b, f: 200 μm, c-d, e, h: 50 μm, g:25 μm

**Figure 4:**
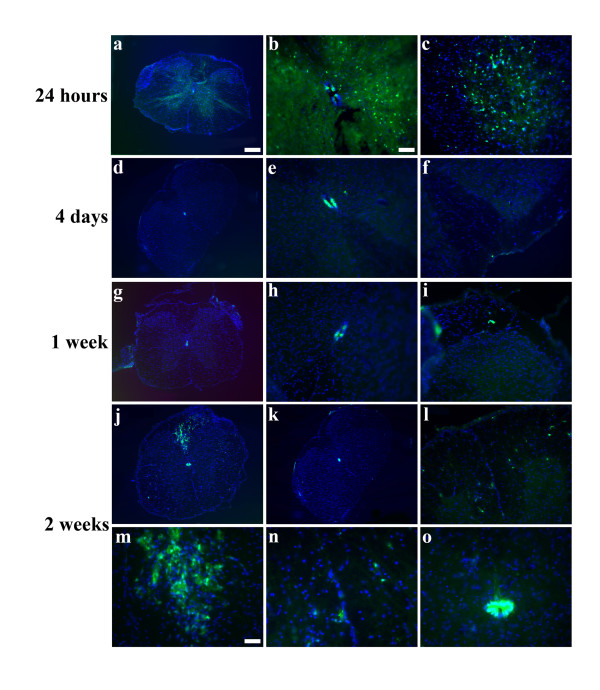
**CXCR4-GFP cells appear in the periphery and in the dorsal funiculus in 2 weeks**. At 24 hours post injury, CXCR4-GFP cells appear throughout the gray and white matter, but this expression is gone by 4 days post injury(a-f). The ependymal layer, which was full of CXCR4-GFP prior to injury, has fewer CXCR4-GFP cells post injury (a-b, d-e, g-h, j-k). A sprinkling of CXCR4-GFP cells are seen toward the meninges at 1 week(g-i), but the majority of the 2 apparently migrating populations arrive by 2 weeks(j, l-o). Caudal to the injury, no cells are again seen in the dorsal funiculus(k).(green:CXCR4-EGFP, blue:Hoechst). Scale bars: a, d, g, j-k: 200 μm, b-c, e-f, h-I, k-l: 50 μm, m-o: 25 μm.

The pattern of CXCR4 expression changed dramatically in the spinal cord 5 weeks following SCI. CXCR4-GFP+ cells continued to be present in the ependymal layer, although there appeared to be fewer than in the uninjured spinal cord (Figure 3a-d, Figure 4a-b, d-e, g-h, j-k). However there were also numerous CXCR4+ cells that appeared to be migrating towards the periphery of the spinal cord, possibly towards the SDF1 expressed by the meninges. CXCR4-GFP+ cells were also present in the dorsal funiculus of the spinal cord, located between the dCST and the gray matter, but only rostral to the injury where SDF1 continued to be present. Although occasional CXCR4+ cells resided within the lesion epicenter and scar, most of them resided outside of it (Figure 3f-h). CXCR4 and GFAP expression did not overlap at all within the scar indicating that astrocytes do not express the receptor (Figure 3g).

### Timecourse of CXCR4 expression post injury

To better understand the changes in the CXCR4 expression pattern post injury, we examined the time course of CXCR4 expression. At 24 hours post injury, we observed numerous CXCR4+ cells scattered throughout the gray and white matter, possibly reflecting the well-described microglial activation that occurs immediately following SCI (Figure 4a-c). By 4 days, however, this activation was attenuated and very few peripheral CXCR4+ cells were present at 1 week (Figure 4d-i). By 2 weeks, however, many more CXCR4+ cells appeared all around the periphery of the spinal cord in closed juxtaposition to the meninges and in the dorsal funiculus rostral to the lesion (Figure 4j, l-0). Importantly, the dorsal funiculus caudal to the lesion again had no CXCR4+ cells (Figure 4k). Throughout the timeline, CXCR4 expression persisted, but the staining was progressively somewhat attenuated in the ependymal layer

### Identification of the CXCR4+ cell types

**Figure 5:**
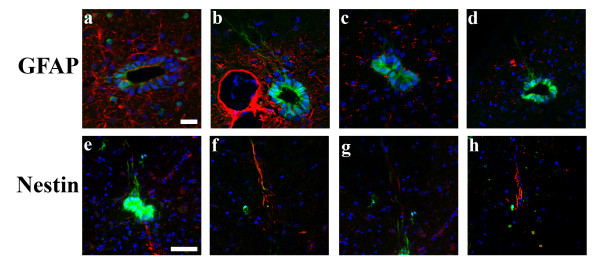
**CXCR4-GFP cells in the ependymal layer do not have neural progenitor markers**. The CXCR4-GFP cells in the ependymal layer have long extended processes, but do not colocalize with GFAP+ processes at 24 hours post injury (a-b) or at 2 weeks (c-d)(red:GFAP, green:CXCR4-EGFP, blue:Hoechst). At 2 weeks, Nestin+ processes also extend from the ependymal layer, but do not colocalize with CXCR4-GFP(e-h). Occasionally, however, the Nestin+ processes are in close apposition to CXCR4-GFP processes (f) (red:Nestin, green:CXCR4-EGFP, blue:Hoechst). Scale bars: a-d: 20 μm, e-h: 50 μm.

**Figure 6:**
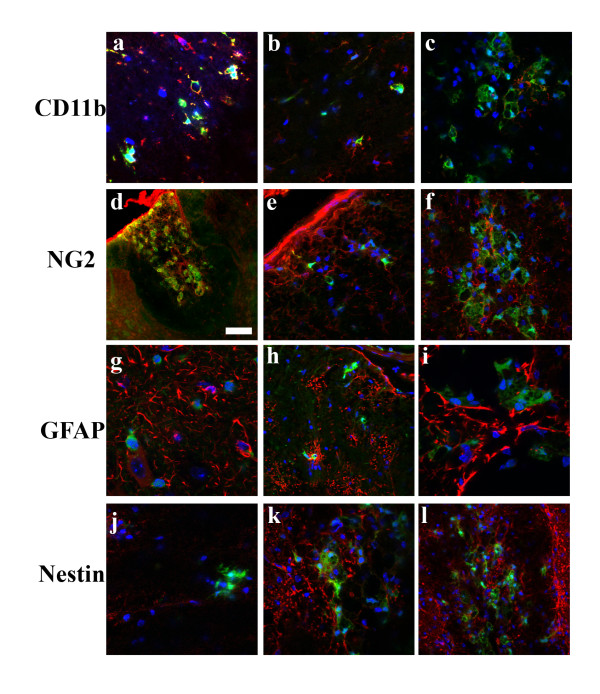
**CXCR4-GFP cells in the general periphery and in the dorsal funiculus express markers of hematopoetic lineage and not neural lineage**. At 5 weeks (a, d) and at 2 weeks (b-c, e-f), the putative migrating cell populations are positive for both CD11b and NG2 identifying them as presumptive macrophages. No CXCR4-GFP cells colocalized with either GFAP or Nestin. Scale bars: d: 50 μm. [red:CD11b(a-c), NG2(d-f), GFAP(g-i), Nestin(j-l), green:CXCR4-EGFP, blue:Hoechst].

The number of different cell types in the ependymal layer of the spinal cord is unknown, and their functional phenotypes, especially with respect to their potential as stem/progenitor cells, remain unclear [28-33]. We used the CXCR4-EGFP reporter mouse to help identify specific subsets of cells in the ependymal layer. First we looked for CXCR4+ neural progenitors by co-staining for the neural markers, GFAP and Nestin. Nestin and GFAP both labeled cells in the ependyma and processes extending from the ependymal layer, but neither colabeled with CXCR4 (Figure 5). The CXCR4+ processes also did not colocalize with RC2 that is a marker for radial glial progenitors (data not shown). However Nestin+ fibers sometimes abutted CXCR4+ fibers indicating a close association (Figure 5f). We also looked for potential CXCR4+ progenitors that had migrated out toward the periphery, but we did not see any colocalization of GFAP+ or Nestin+ with the CXCR4+ peripheral cells. However, CXCR4 did colocalize with CD11b and NG2 suggesting that these cells are infiltrating macrophages (Figure 6). By contrast, the CXCR4+ ependymal cells were not positive for CD11b or NG2 (data not shown). These observations suggest that there are at least two distinct populations of CXCR4+ cells after SCI, ependymal cells and migrating macrophages.

## Discussion

### SDF1 is expressed in dCST axons

Although SDF1 has been suggested as being important in CST development[11], its cellular origins have not been clarified in either the normal or the injured adult spinal cord. Shechter et al described SDF1 in the dorsal horn of the spinal cord, but their immunohistochemistry associated SDF1 with GFAP[20]. However the availability of SDF1-EGFP transgenic reporter mice allowed us to more precisely localize the chemokine, and we never found the chemokine to be co-expressed with GFAP. However we did find that SDF1 was consistently expressed by neurons contributing to the main dCST bundle of the CST but not the dorsolateral or ventral tracts. Tract tracing studies confirmed the cellular identity of the SDF1-containing fibers in the spinal cord (Figure 1e, f, g). This expression pattern is consistent with the observations of Schonemeier et al (2008) that SDF-1 was expressed by neurons in layer V of the cortex.

### Function of SDF1 in the spinal cord

The expression of SDF1 in CST neurons suggests that it might have a neurotransmitter of similar function in the spinal cord. Indeed, SDF1 has been shown to act as a neuromodulator in some neural systems such as the hippocampus [23], and other studies have reported an upregulation of SDF1 post CNS disease [19]. SDF-1 has been shown to be sufficient to overcome neurite outgrowth inhibition mediated by CNS myelin towards cultured postnatal dorsal root ganglion neurons. Further, local intrathecal infusion of SDF-1 following thoracic dorsal hemisection resulted in enhanced sprouting of corticospinal tract axons into white and grey matter [34]. However, since we find that CST neurons do not express CXCR4, it seems likely that other cells that express the receptor indirectly mediated the effects of SDF1 infusion. This correlates well with our observation that CXCR4+ cells appeared to migrate towards the injured spinal cord. Our observations thus suggest that the injured CST releases SDF1 that attracts macrophages and perhaps other cells that help to stimulate CST axon regrowth.

### CXCR4+ cells migrate specifically to the dorsal funiculus after SCI

The ependymal layer in the spinal cord has been reported to supply progenitors similar to the ventricular layer in the brain, augmenting general interest in the ependymal layer [28-33]. Several groups have implicated SDF1/CXCR4 signaling in the migration of CXCR4+ progenitors post injury [19-21]. However, Meletis et al deemed it unlikely that CXCR4 caused the migration of progenitors toward injury since they saw only a few ependymal cells expressing CXCR4 [28]. Our model allowed us to easily identify cells expressing CXCR4 and we observed numerous CXCR4+ cells in the ependyma. In fact, the ependymal layer appears to express CXCR4 uniformly although this does decline somewhat subsequent to injury. One hypothesis is that neural progenitor cells migrate from the ependymal layer possibly using CXCR4 as their sensor toward areas of injury. In fact changes in levels of SDF1 after spinal cord injury were associated with changes in numbers of CD133-expressing ependymal pericytes, suggesting a possible role in regulating ependymal stem/progenitor cells [35]. Although our static studies did not allow us to study the migration of cells away from the ependymal zone, we frequently observed CXCR4+ cells that appeared to be migrating in this fashion. However we did not find any CXCR4+ cells that had markers of neural lineages. There are several possible explanations for this. Progenitor cells are a rarer cell population than infiltrating cells post SCI and we may not be catching them with our analysis. Alternatively, progenitor cells may migrate under the influence of CXCR4 signaling and then differentiate. CXCR4+ ependymal cells themselves may not migrate away from the lesion, but may simply downregulate CXCR4 in response to other signals in the environment.

### Identity of most CXCR4+ cells in the spinal cord post injury

The major component of the two migrating populations of CXCR4+ cells post injury seem to come from the same lineage. They are both infiltrating cells passing through the lesion and migrating within 2 weeks toward sources of SDF1, be it toward the meninges alone, or in between two sources of SDF1, the meninges and the dCST. Most of these CXCR4 expressing cells also express CD11b and NG2 identifying them as hematopoetic lineage cells, most likely macrophages [36]. The role of macrophages after SCI is controversial, and they have been posited to have both beneficial and deleterious effects[37,38]. The high expression of SDF1 by the dCST and the apparent release of the chemokine after injury suggest that attraction of CXCR4+ macrophages is part of a programmed response to injury. In turn this suggests that that modulation of the SDF1 signaling system may be important for regulating the inflammatory response after SCI.

## Conclusions

In the adult uninjured spinal cord, SDF1 and CXCR4 are expressed in separate, nonadjacent regions: meninges and dCST vs. the ependymal layer. Following the breach in blood brain barrier post severe SCI, multiple sources of both proteins exist contributing to specific post-injury signaling. CXCR4-expressing macrophages migrate through the spinal cord toward sources of SDF1 and arrive in the peripheral spinal cord, toward the SDF1 in the meninges, and toward the intact dCST only rostral of the injury. This migration occurs between 1 and 2 weeks post injury. CXCR4-expressing ependymal cells remain post injury, but are in fewer number than in the uninjured spinal cord. Therefore, CXCR4 and SDF1 signaling does introduce a trauma-specific response post SCI and may then have a specific role.

## Competing interests

The authors declare that they have no competing interests.

## Authors' contributions

VT led the study and performed most of the tissue processing, immunohistochemistry, and all of the data analysis. DM and HJ bred animals for the study and participated in its design. VS and DB assisted with the surgeries, the animal care, and participated in the study's design. The reporter mice were generously donated by RM's lab. VT, RM, and JK prepared the manuscript. All authors read and approved the final manuscript.
