# SDF1 in the dorsal corticospinal tract promotes CXCR4+ cell migration after spinal cord injury

**DOI:** 10.1186/1742-2094-8-37

**Published:** 2011-04-20

**Authors:** Vicki M Tysseling, Divakar Mithal, Vibhu Sahni, Derin Birch, Hosung Jung, Abdelhak Belmadani, Richard J Miller, John A Kessler

**Affiliations:** 1 Northwestern University's Feinberg School of Medicine, Department of Neurology, Chicago, IL 60611, USA; 2 Northwestern University's Feinberg School of Medicine, Department of Molecular Pharmacology and Biological Chemistry, Chicago, IL 60611, USA

## 

The original article [1] was initially published with the following list of authors: Vicki M Tysseling, Divakar Mithal, Vibhu Sahni, Derin Birch, Hosung Jung, Richard J Miller, and John A Kessler. This author list is now corrected as follows: Vicki M Tysseling, Divakar Mithal, Vibhu Sahni, Derin Birch, Hosung Jung, Abdelhak Belmadani, Richard J Miller, and John A Kessler). Abdelhak Belmadani has been added as co-author to this corrected author list.
