# Erratum to: SDF1 in the dorsal corticospinal tract promotes CXCR4+ cell migration after spinal cord injury

**DOI:** 10.1186/s12974-017-0810-0

**Published:** 2017-02-14

**Authors:** Vicki M. Tysseling, Divakar S. Mithal, Vibhu Sahni, Derin Birch, Hosung Jung, Richard J. Miller, John A. Kessler

**Affiliations:** 1 Department of Neurology, Northwestern University’s Feinberg School of Medicine, Chicago, IL 60611 USA; 2 Department of Molecular Pharmacology and Biological Chemistry, Northwestern University’s Feinberg School of Medicine, Chicago, IL 60611 USA

## Erratum

During production of the original article [1], Divakar Singh Mithal's middle initial was omitted, and listed as 'Mithal D'. The correct attribution is 'Mithal DS'.
